# Complete genome sequence of *Propionimicrobium* sp. PCR01-08-3, isolated from the feces of the *Tenebrio molitor* larvae fed with styrofoam

**DOI:** 10.1128/mra.00824-25

**Published:** 2025-10-31

**Authors:** Lintao Wu, Guowen Dong, Jingjing Zhao, Yin Li, Chih-Hung Wu, Wangchuan Xiao, Shu-Jung Lai, Yen-Chi Wu, Chao-Jen Shih, Wei-Ling Zhang, Song Wang, Wanling Qiu, Mengyan Han, Hangying Zhang, Sheng-Chung Chen

**Affiliations:** 1 College of Resources and Environment, Fujian Agriculture and Forestry University 602381, Fuzhou, Fujian, People’s Republic of China; 2 School of Resources and Chemical Engineering, Sanming University 659925, Sanming, Fujian, People’s Republic of China; 3 Fujian Provincial Key Laboratory of Resources and Environmental Monitoring and Sustainable Management and Utilization, Sanming University 66283, Sanming City, Fujian, People’s Republic of China; 4 School of Chemistry and Materials, Fujian Normal University 12425, Fuzhou, Fujian, People’s Republic of China; 5 College of Environment and Safety Engineering, Fuzhou University 562589, Fuzhou, Fujian, People’s Republic of China; 6 Graduate Institute of Biomedical Sciences, China Medical University, Taichung City, Taiwan, Republic of China; 7 Research Center for Cancer Biology, China Medical University, Taichung City, Taiwan, Republic of China; 8 Bioresource Collection and Research Center, Food Industry Research and Development Institute, Hsinchu, Taiwan, Republic of China; University of Southern California, Los Angeles, California, USA

**Keywords:** gut microbiota, *Tenebrio molitor*, anaerobes, *Propionimicrobium*, styrofoam

## Abstract

The gut microbiota of *Tenebrio molitor* has attracted attention for its potential in solving plastic waste problems. Here, we report the complete genome sequence of *Propionimicrobium* sp. PCR01-08-3, isolated from the feces of *Tenebrio molitor* larvae. The genome of strain PCR01-08-3 was selected for further species delineation and comparative genomic analysis.

## ANNOUNCEMENT

**Fig 1:**
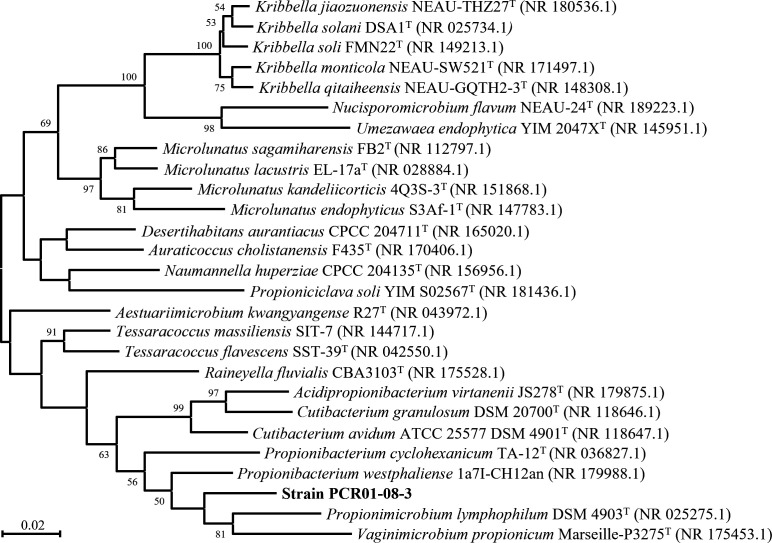
Maximum likelihood tree based on 16S rRNA gene sequences of strain PCR01-08-3 and related taxa. Sequence alignment was performed using Clustal W (7), and the Tamura-Nei model (8) was applied as the nucleotide substitution model. Bar, 0.02 substitutions per nucleotide position. Bootstrap values were expressed as percentages of 1,000 replicates.

Strain PCR01-08-3 was isolated from the feces of *Tenebrio molitor* larvae that had been continuously fed with polystyrene at ~25°C for over 6 months. Approximately 100 larvae were originally obtained from “Pan Nongjia Manor,” an online supplier in Shaoxing, Zhejiang, China. Fresh feces were inoculated into modified anaerobic DSM 1523 medium (N_2_:CO_2_ = 4:1, without 1 g/L acetate and 2 g/L formate) at ~25°C for 2 weeks. To further purify and identify the strain PCR01-08-3, a combined approach was employed, integrating serial dilution, rolling-tube technique (1), single-colony isolation, and colony PCR-based 16S rRNA gene clone sequencing using 8F and 1492RU primers (2). Bacterial purity was confirmed through morphological observation, 16S rRNA gene sequencing, and genome sequencing. Based on the analysis through BLASTN (3), strain PCR01-08-3 showed the highest similarities to both characterized strains, *Propionibacterium westphaliense* 1a7I-CH12an (NR_179988, 94.69%) (4) and *Propionimicrobium lymphophilum* DSM 4903^T^ (NR_180133, 94.07%) (5). Phylogenetic analysis of 16S rRNA gene sequences performed by MEGAX (6) for strain PCR01-08-3 and related taxa indicated that strain PCR01-08-3 could be a novel lineage (Fig. 1).

The strain PCR01-08-3 was grown in modified DSM 1523 medium and incubated at ~25°C for 3 days until the stationary phase. The genomic DNA of strain PCR01-08-3 was extracted using the QIAGEN Genomic-tip 20/G kit according to the manufacturer’s instructions. The DNA was quantified and qualified using a NanoDrop 2000 spectrophotometer and Qubit TM3 fluorometer (Thermo Fisher Scientific). Library preparation involved shearing 15 µg of DNA with g-TUBE (Covaris) according to the manufacturer’s protocol to achieve a target fragment size of ~15 kb, as required for PacBio long-read sequencing. Small fragments (<1 kb) were removed using AMPure PB Beads to enrich larger fragments and improve library quality. The SMRTbell library was constructed following the instructions of the SMRTbell Express Template Preparation Kit 2.0 (PacBio, Cat No. 100-938-900) and sequenced at the Biomarker Technologies (BMKGENE) Co., Ltd. (Beijing) using the PacBio Sequel II sequencer (v2.0 chemistry, Pacific Biosciences).

Raw subreads obtained from the PacBio Sequel II platform were processed using the SMRT Link v10.1 (Pacific Biosciences) to generate circular consensus sequences. Low-quality and short reads (<2,000 bp) were filtered out, and the resulting 63,099 high-quality reads (N_50_, 8,561 bp) were used for subsequent *de novo* assembly. Genome assembly was carried out using Hifiasm v0.16.1 (9). Circularization of the genome was verified using Circlator v1.5.5 (10), which identified overlapping ends and confirmed complete circular topology and was polished with Pilon v1.22 (11). Contigs shorter than 1 Mb were preliminarily regarded as plasmids. The result yielded a genome of 3,248,514 bp (63.43% GC) and a plasmid of 40,493 bp (61.08% GC). The NCBI Prokaryotic Genome Annotation Pipeline (PGAP, v6.7) was used for functional annotation (12). The genome was predicted to have 3,060 genes, of which 2,965 were protein-coding, and contains 6 rRNA genes and 45 tRNA genes. Default parameters were applied in all bioinformatics analyses.

Genome annotation identified several oxidative and hydrolytic enzyme genes, including a Dyp-type peroxidase (WP_286026142.1), cytochrome P450 monooxygenases (WP_286024933.1 and WP_286026257.1), an acetylxylan esterase (WP_286024579.1), and an α/β-hydrolase (WP_286026680.1), which may participate in the degradation or transformation of polystyrene-derived intermediates.

## Data Availability

The sequences of the genome and plasmid of strain PCR01-08-3 have been deposited in GenBank under accession numbers CP127390.1 and CP127391.1, respectively. The version of the assemblies described in this paper is the first version. The BioProject and BioSample accession numbers are PRJNA981099 and SAMN35661488, respectively. The raw sequence reads were deposited in the Sequence Read Archive (SRA) under accession number SRR24928164.
